# Deep Reinforcement Learning for Workload Prediction in Federated Cloud Environments

**DOI:** 10.3390/s23156911

**Published:** 2023-08-03

**Authors:** Zaakki Ahamed, Maher Khemakhem, Fathy Eassa, Fawaz Alsolami, Abdullah Basuhail, Kamal Jambi

**Affiliations:** Department of Computer Science, Faculty of Computing and Information Technology, King Abdulaziz University (KAU), Jeddah 21589, Saudi Arabia; makhemakhem@kau.edu.sa (M.K.); feassa@kau.edu.sa (F.E.); falsolami1@kau.edu.sa (F.A.); abasuhail@kau.edu.sa (A.B.); kjambi@kau.edu.sa (K.J.)

**Keywords:** Deep Reinforcement Learning, Deep Q learning, workload prediction, Federated Cloud Computing, energy efficiency, Virtual Machine placement, Machine Learning

## Abstract

The Federated Cloud Computing (FCC) paradigm provides scalability advantages to Cloud Service Providers (CSP) in preserving their Service Level Agreement (SLA) as opposed to single Data Centers (DC). However, existing research has primarily focused on Virtual Machine (VM) placement, with less emphasis on energy efficiency and SLA adherence. In this paper, we propose a novel solution, Federated Cloud Workload Prediction with Deep Q-Learning (FEDQWP). Our solution addresses the complex VM placement problem, energy efficiency, and SLA preservation, making it comprehensive and beneficial for CSPs. By leveraging the capabilities of deep learning, our FEDQWP model extracts underlying patterns and optimizes resource allocation. Real-world workloads are extensively evaluated to demonstrate the efficacy of our approach compared to existing solutions. The results show that our DQL model outperforms other algorithms in terms of CPU utilization, migration time, finished tasks, energy consumption, and SLA violations. Specifically, our QLearning model achieves efficient CPU utilization with a median value of 29.02, completes migrations in an average of 0.31 units, finishes an average of 699 tasks, consumes the least energy with an average of 1.85 kWh, and exhibits the lowest number of SLA violations with an average of 0.03 violations proportionally. These quantitative results highlight the superiority of our proposed method in optimizing performance in FCC environments.

## 1. Introduction

The Cloud computing paradigm has revolutionized the way the IT industry operates due to its ease of use, scalability, and the ability to offer flexible services such as Software-as-a-Service (SaaS), Platform-as-a-Service (PaaS), and Infrastructure-as-a-Service (IaaS) [1,2]. This presents a wide range of options for organizations. However, with the wide-scale adoption of this paradigm, the challenge of keeping up with user demand is of high priority. When the user demands outpace the available resources, the organization loses business due to violation of the Service Level Agreement (SLA) [3].

The Federated Cloud Computing (FCC) paradigm introduces a novel solution to this challenge by enabling resource sharing among Cloud Service Providers (CSPs). When a certain CSP is unable to accommodate the resource requirements of a user, it offloads the work to another federation member CSP with available resources to process it [4,5]. This way, FCC allows CSPs to accommodate more users or handle larger workloads than they would be able to with a single  Data Center (DC).

However, this federated approach introduces a complex problem of efficiently allocating resources, considering all the DCs within the federation. This is due to the fact that DCs in an FCC environment can vary significantly in terms of hardware configurations, network capabilities, geographic locations, and service offerings. The resource allocation decisions must consider SLA requirements at each DC, ensuring that service commitments are met while optimizing resource utilization, avoiding bottlenecks and ensuring responsiveness for time-critical tasks. Moreover, the high energy consumption of cloud computing has a significant environmental impact [6,7], making it crucial for CSPs to minimize energy consumption and improve efficiency to enhance their profitability.

While existing literature has explored resource management in cloud environments, with a focus on Virtual Machine (VM) placement [8,9], the research on energy efficiency and SLA preservation in the context of FCC environments has been relatively limited. With the recent boom in Machine Learning (ML), particularly Deep Learning (DL), the adoption of DL techniques has become commonplace for optimizing complex multi-dimensional problems in this domain [10,11,12,13]. In this paper, we propose a detailed analysis of our novel solution, Federated Cloud Workload Prediction with Deep Q-Learning (FEDQWP) for workload prediction in FCC environments using Deep Q Learning (DQL). It addresses the challenges of VM placement, energy efficiency, and SLA preservation. By incorporating key metrics such as energy consumption, SLA violations, VM migrations, and migration durations, our solution offers a comprehensive approach. Furthermore, we evaluate our proposed solution using real-world workloads and compare its efficacy with other state-of-the-art solutions in the domain.

Accordingly, this research aims to address the following research questions:

1. How can DQL be implemented as an optimal VM scheduling and energy-efficient strategy while respecting SLA agreements in an FCC environment?
2. What is the overall impact of using DL in solving complicated multi-dimensional problems for system performance?
3. How does the proposed novel solution perform compared to existing solutions in terms of energy efficiency, SLA adherence, and cost-effectiveness?
4. How can the proposed solution be extended to handle dynamic workloads and changing resource availability in FCC environments?
5. What are the associated challenges and trade-offs of implementing our solution?

The main contributions of this article include:

- A novel solution, FEDQWP, to the problem of optimizing VM placement and energy efficiency while preserving SLA in FCC environments using DQL.
- A comprehensive evaluation of the proposed solution using real-world workloads, demonstrating its effectiveness compared to existing solutions in the domain.
- A deeper understanding of the benefits and challenges of using DQL in optimizing complex multi-dimensional problems.
- A framework for handling dynamic workloads and changing resource availability in FCC environments.

It is expected that the proposed solution provides a significant contribution to the field of cloud computing by addressing key questions and challenges and providing new directions for potential research. The subsequent sections of this article are structured as follows: Section 2 presents a comparative analysis of the existing literature. Section 3 discusses the architecture of the federation and the operations of FEDQWP. Section 4 explains the findings of the analysis. Section 5 details the interpretation of the results, and the paper concludes in Section 6. Section 7 discusses possible future directions.

## 2. Related Works

Much research has been conducted in the field of predicting and allocating resources in the Cloud Computing environment. A Deep Belief Network (DBN) model proposed by Qiu et al. [10] consists of a multi-layer architecture that is capable of extracting high-level features from the historic load data and the logical regression layer using Restricted Boltzmann Machines (RBM). The designed model can be used to forecast the workload for a single VM or for multiple VMs. The input layer at the bottom is where the Central Processing Unit (CPU) utilization data are fed, and the top layer provides the predicted output. While the accuracy of the CPU utilization prediction is improved, the resources needed for training are high. Moreover, the authors have not incorporated the prediction with other parts of the VM consolidation algorithm.

Beloglazov et al. [14] focused on analyzing past resource usage data using adaptive heuristics for VM consolidation. They employed variations of the Local Regression algorithms to predict the CPU utilization of hosts. This in combination with a Minimum Migration Time (MMT) approach has been put forth as a power-aware model which takes into account the level of SLA violation and the number of VM transfers. While the comparison shows that it provides better results, it does not explain the high variations, where in some instances, high energy consumption results in no SLA violations and low energy consumption results in high SLA violations.

Kumar et al. [15] have combined the Long Short Term Memory (LSTM) model and Recurrent Neural Network (RNN) models to solve the issue of dynamic resource scaling in a cloud environment. They put forth the argument that the best way to predict resource consumption is to learn the patterns from historical data. While RNNs have the ability to extract that information, when time series problems are concerned, they do not perform well over longer durations. Combining this with LSTMs balances this downside as LSTMs preserve information over longer periods. The predicted output from the proposed design is then fed into the Resource Manager (RM) that measures the present condition of the DC, which facilitates it to scale up or scale down the resources accordingly. Even though it gives higher accuracy, the amount of data for it to be effective and the computational resources for training might be prohibitive for it to be effective.

Zhu et al. [16] propose a novel solution involving the attention mechanism along with the LSTM encoder-decoder pairings. Initially, the scheme extracts the sequential features of the past utilization data using the encoder network. For the next phase, the attention mechanism is employed in the decoder network for batch workload prediction. The context vector takes in the input workload for the encoding phase and the decoder recursively unravels the context vector providing an output of the predicted sequence. This method is effective in mitigating error amplification and improving accuracy for longer-duration prediction. However, more resources are needed for processing a larger model, as more epochs are needed to converge. This is a result of it being costlier than training single-layer models.

A clustering-based workload prediction method is proposed by Gao et al. [17]. This is so that all workload patterns are captured covering heterogeneous tasks. Two kinds of clustering techniques were used: Prototype-Based Clustering (PBC) and Density-based clustering (DBC). Furthermore, it revolves around an approach named m-gap prediction that buffers a gap of specific (m) points of time between input data and the forecasted data points. This allows sufficient time for task scheduling. This, in combination with clustering, pools similar workload patterns into classes which are stored as a model which in turn is used for the final prediction. Even though the prediction accuracy is good, this approach does not scale well to larger datasets and the user has to explicitly specify the number of clusters.

The Savitzky–Golay and Wavelet-supported Stochastic Configuration Networks (SGW-SCN) framework is proposed by Bi et al. [18] as an integrated machine-learning solution for DCs. It utilizes the SGW filter used to eliminate the noise while preserving the width and peaks of the signal. It also uses the Haar wavelet method to extract the patterns from time series data. Furthermore, it performs well with various scales of resolution. The SCN is a supervised learning method using the randomized learning model for higher accuracy. Thus, it increases the learning speed of the model. However, the tradeoff is a higher resource consumption for the training of the model.

All of the aforementioned works attempt to deal with workloads of high variance and high dimensional space [19]. This forces the model to grapple with more complex data to come up with a solution. FEDQWP builds on these works and is distinguished by considering both VM placement and energy efficiency while preserving SLA agreements using DQL in FCC environments. The draw of Q Learning is that they are not as expensive computationally to train and deploy as opposed to the above mentioned instances [20]. No existing solutions comprehensively tackle the challenges mentioned previously, to the best of our knowledge. FEDQWP represents a significant improvement over existing solutions in the domain, demonstrating the potential of DQL in solving complex multi-dimensional problems in FCC environments.

## 3. Methodology

### 3.1. Proposed Cloud Federation Architecture

Even with so many organizations investing in Cloud Computing technology, there is a finite capacity when it comes to providing resources. To move beyond this limitation, the best approach would be to collaborate and share resources. The applications offered in a CSP should be virtually contained within a VM to be migrated between different CSPs. This pooling of resources is a win-win scenario for all parties involved—the user receives uninterrupted service due to the processing request being offloaded, the CSP associated with the user does not violate the SLA and does not over-extend its capacity, and the CSP handling the offloaded request is able to utilize idle computing power to make extra income.

**Figure 1:**
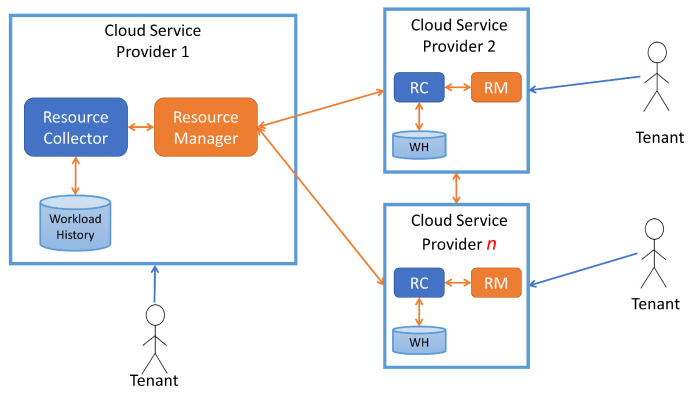
Architecture of the Federation.

As shown in Figure 1, there will be multiple CSPs. In our case, we will be working with a federation of 3 CSPs. Each CSP has an RM unit that hosts the components of the FEDQWP including the Deep Reinforcement Learning (DRL) agents along with the resource scaling functionalities. The Resource Collector (RC) interfaces with the manager to collect the workload history and store it for future use. We assume that:

- The CSPs are de-centralized and operate on a peer-to-peer basis.
- The RM functions as the resource broker for each CSP.
- Each resource broker has its ledger of available resources.

The user request is packaged into VMs which will be transferred among the various CSPs in the federation.

**Figure 2:**
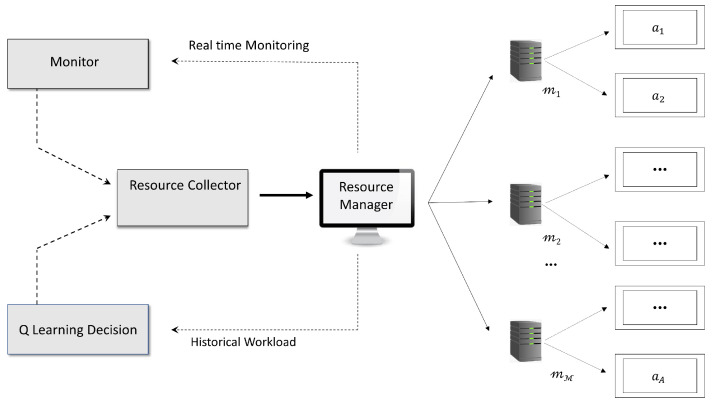
Architecture of the Resource Manager.

The vital part of the architecture is the RM. As shown in Figure 2, the Resource Manager is connected to the hosts numbered from $m_{1}$ to $m_{M}$. Each of the hosts executes the applications inside specific VMs which are isolated from each other and denoted by $a_{1}$ to $a_{A}$. Processing requests are sent by consumers of the CSP. Each request occupies one VM container which occupies space within a host along with other similar VM containers. The RM keeps track of the activities of the consumer, the time taken to process the request, and the power consumption of the VMs, and determines how the VM containers are moved within the CSPs.

### 3.2. Q Learning Algorithm Implementation

Our solution to the optimal migration problem is a DQL algorithm to learn the best policy for predicting the workload and scheduling the VM accordingly. The model employs an agent which regularly interacts with and samples the condition of the environment. The agent is aware of the nature of the workload and the energy consumption parameters and makes decisions accordingly for VM placement. Depending on the feedback based on the action taken, the agent is rewarded or penalized accordingly on how the SLA compliance and energy consumption are affected.

The DRL model is trained using a combination of deep Neural Networks (NN) and DRL algorithms. We use a deep NN to approximate the optimal policy, and we use the Q-learning algorithm to update the Q-values of the state-action pairs. The learning agents learns the optimal decisions by interacting with the environment on a “trial-and-error” based approach. This rule means the DRL agents make a trade-off between known decision exploitation and new decision exploration to achieve the optimal policy.

**Figure 3:**
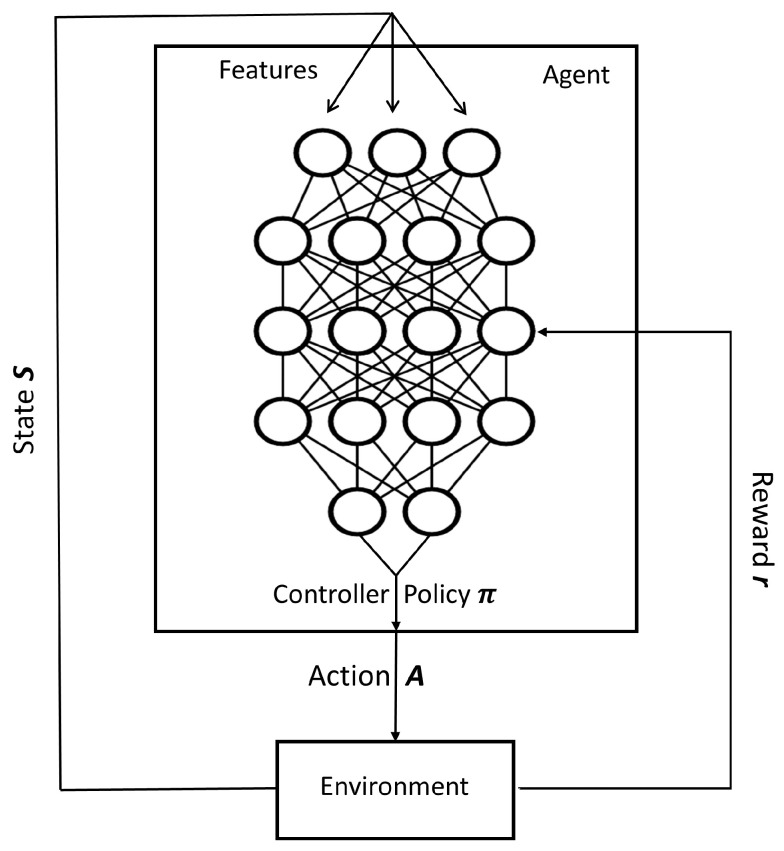
Architecture of the Deep Q Learning Algorithm.

As is shown in Figure 3, our architecture relies on:

- **S**: The finite set S includes the possible states of the environment. The state space includes various features extracted from the collected data, such as the current workload, resource utilization, and energy consumption.
- **A**: The finite set S(*s*) is the set of actions available in state *s*. The action space includes the set of possible actions that can be taken, such as adding or removing VMs, migrating VMs between data centers, and adjusting resource allocation.
- π: The policy that maps from S and action A. π (*s*, *a*) denotes the probability of acting under state *s*.
- ***r***: The reward function is used to evaluate the goodness of an action taken by the agent. In our case, the reward function is designed to minimize energy consumption while ensuring SLA compliance. The reward function is defined as a weighted sum of delay, power consumption and migration cost, where the weights are determined based on the importance of each objective.

The SLA is determined based primarily on the response time. The response times of migrations are sorted in ascending order; then, calculating the value at the 95th percentile, the algorithm determines the threshold response time that 95% of the requests should meet to satisfy the SLA. Any response time exceeding this 95th percentile value would be considered a violation of the SLA.

**Figure 4:**
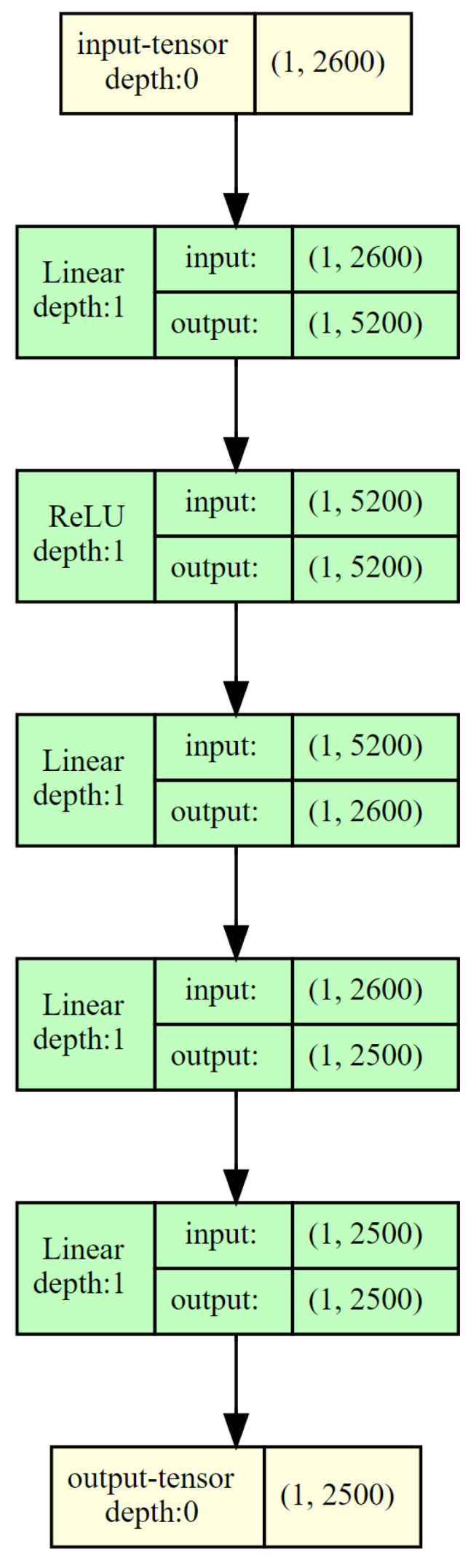
Structure of the Q Learning Neural Network.

The problem of training is tackled by a two-pronged solution. There are two NNs for training and prediction, as illustrated in Figure 4. The input and output layers have 5 hidden layers between them, with 2500 to 5200 nodes for each layer. The ReLu activation function is used to introduce nonlinearity to allow the agent to develop complex representations. The choice of ReLu over other functions such as Sigmoid is due to neurons not being activated at the same time by the former. As a result, it reaches convergence faster than other activation functions [21]. This in combination with the DQL algorithm is utilized for quick decision-making, as workloads have a tendency to fluctuate and decisions have to be taken in real-time [19].

The state of the system is dependent on the migration cost, power consumption, and the delay involved in resolving VMs. The delay is related to the location of the ith host at a given time and the resource requirement of the ith VM at a given time. This is denoted by the following equation:

(1) $$S\left(\phi \right)=\left[\begin{array}{ccc} S_{1,1}\left(\phi \right) & \cdots & S_{1,y}\left(\phi \right)\\ \vdots & \ddots & \vdots \\ S_{x,1}\left(\phi \right) & \cdots & S_{x,y}\left(\phi \right) \end{array}\right]$$

In Equation (1), $S\left(\phi \right)$ is the system state of delay during the ϕth time slice. ϕ is the serial number of the ϕth time slice, where $S_{x,y}\left(\phi \right)$ represents the state of delay of the *i*th node and (*i*th VM container).

To represent the main metrics used, $x_{total}$ denotes the total delay, $y_{total}$ is for the total power consumption and $z_{total}$ is the total computational cost of migration by aggregating the individual delay, power consumption, and migration cost values during a unit of time (slice)—ϕ, where a slice $T_{\phi }$ is enumerated as (ϕ = 0,..., k). It is denoted as:

(2) $$\begin{array}{c} x_{\mathrm{total}}=\sum_{\phi =0}^{\kappa }\left(\sum_{i=1}^{l}x_{{\mathrm{net}}_{i}}\left(\phi \right)+k_{\mathrm{comp}}\times \sum_{i=1}^{n}x_{{\mathrm{comp}}_{i}}\left(\phi \right)\right) \end{array}$$

In Equation (2), $x_{net}$ represents the delay associated with the network between the user and the relevant nodes assigned during a certain time slice. $x_{comp}$ represents the delay in the computation of application tasks. They are confined to the *i*th VM container.

(3) $$y_{\mathrm{total}}=\sum_{\phi =0}^{\kappa }\sum_{i=1}^{m}y_{{\mathrm{total}}_{i}}\left(\phi \right)$$

In Equation (3), $y_{{total}_{i}}\left(\phi \right)$ represents the estimated power consumption of the *i*th node during time slice ϕ.

(4) $$z_{\mathrm{total}}=\sum_{\phi =0}^{\kappa }\sum_{i=1}^{n}z_{{\mathrm{total}}_{i}}\left(\phi \right)$$

In Equation (4), $z_{{\mathrm{total}}_{i}}\left(\phi \right)$ represents the cost of migrating the *i*th VM container during time slice ϕ.

We focus on minimizing the total cost over time; thus, the reward during a specific time slice is defined as:

(5) $$R_{\phi }=-\left(\omega_{1}x_{\mathrm{total}}\left(\phi \right)+\omega_{2}y_{\mathrm{total}}\left(\phi \right)+\omega_{3}z_{\mathrm{total}}\left(\phi \right)\right)$$

In Equation (5), $R_{\phi }$ is the reward given to the agent during the ϕth time slice. $\omega_{1}$ is the weight assigned to the total delay in relation to the impact on the total cost. $\omega_{2}$ represents the weight assigned to the power consumption in relation to the total cost. It is added to the weight of the cost of migration $\omega_{3}$.

### 3.3. DQL Algorithm

The following algorithms provide a step-by-step process for initializing and optimizing the placement of VM containers using our algorithm:

#### 3.3.1. Step 1—VM Container Initialization

In Algorithm 1, first, we initialize new_container_infos to hold information about newly generated VM containers from the workload. Then, we deploy the newly generated VM containers onto the system. The VM containers are created and assigned unique VM container IDs to identify them. The decision for the optimal placement of VM containers is based on the DQL algorithm. Based on the decision, the VM containers migrated for resource optimization. Next, the workload allocation is updated based on the feedback from the simulation. Finally, the current state of the system, including the data center configuration, workload information, scheduler settings, environment variables, and any relevant statistics, are returned.
**Algorithm 1:** Initialization**1** Initialize *new_container_infos* ← workload generates new VM containers from workload;**2** Deploy new VM containers ← Deploy new VM containers and obtain VM container IDs;**3** Decision for placement ← Decide placement using VM container IDs;**4** Migrate VM containers ← Schedule VM containers based on decision;**5** Update ← Update workload allocated using creation IDs;**6** Print ← Agent Decision and Migrations;**7** **return** datacenter, workload, scheduler, env, stats;

#### 3.3.2. Step 2—Agent Initialization

In Algorithm 2, input_size is calculated as the sum of two terms. The first term, 2 * num_VM containers, represents the number of elements in the delay matrix. It is multiplied by 2 because each element in the delay matrix may have two possible values (e.g., minimum delay and maximum delay). The second term, num_hosts * num_VM containers, represents the number of VM containers multiplied by the number of hosts. This part accounts for the states related to mapping VM containers to hosts. Overall, input_size represents the total number of possible states in the environment. Similarly, output_size is determined as the product of the number of hosts (num_hosts) and the number of VM containers (num_VM containers). This represents the number of possible actions the agent can take, which corresponds to the different ways of mapping VM containers to hosts. Finally, the code creates an instance of the Agent class using the calculated input_size, output_size, and other hyperparameters such as learning_rate, gamma, epsilon, epsilon_decay, min_epsilon, replay_memory_size, and batch_size|. This agent will be responsible for learning and making decisions in the cloud environment using the deep Q-learning algorithm.    
**Algorithm 2:** Agent Initialization for DQL**1** input_size ← 2 × num_VM containers + num_hosts × num_VM containers;**2** // Possible states: number of elements in delay matrix + number of VM containers × 2;**3** output_size ← num_hosts × num_VM containers;**4** // Possible actions: number of VM containers × number of hosts;**5** Agent ← input_size, output_size, learning_rate, gamma, epsilon, epsilon_decay, min_epsilon, replay_memory_size, batch_size

#### 3.3.3. Step 3—VM Placement

Algorithm 3 starts by initializing the decision array to store the VM placement decisions. It then iterates over each VM container in the VM container list. If the VM container is None, indicating no VM to place, it moves to the next iteration. Otherwise, the algorithm assigns the VM container ID to variable c. If c is equal to the selected VM container based on the agent’s action, the algorithm appends the VM container ID and the selected host to the decision array. If c is different from the selected VM container, a random host index is chosen, and the VM container ID and the randomly selected host are appended to the decision array. After making the placement decision, the algorithm takes a step in the simulator, passing the selected host and selected VM container values to obtain the next state, reward, and done flag. These values are then appended to the replay memory. The total reward is incremented by the received reward, and the state is updated with the next state for the next iteration. Finally, the algorithm calculates the Q values and expected Q values, updates the loss function, adjusts the epsilon value, increments the episode counter, and outputs the episode count, total reward, and epsilon value.   
**Algorithm 3:** VM Placement in Host for DQL  **1** **Input**: containerlist, selected_container, selected_host, host_list **Output**: decision  **2** Initialize decision array;  **3** **for** *VM_container in containerlist* **do**  **4**  **if** *the VM container is None* **then**  **5**   continue;  **6**  **end**  **7**  c ← VM container_id;  **8**  **if** *c = selected_container* **then**  **9**   decision ← append c and selected_host;**10**  **end****11**  **else****12**   host ← random integer between 0 and length of host_list −1;**13**   decision ← append c and host;**14**  **end****15** **end****16** Assign values for next_state, reward, done ← Take a step in the simulator while passing selected_host, selected_container values;**17** Append replay_memory ← state, action, reward, next_state, done values;**18** Increment total_reward with reward;**19** Update state value with the next state;**20** SET Q Values ← Set state values to the prediction model;**21** SET Target Q Values ← Set target model values to next_state;**22** SET Expected Q Values ← aggregate reward * gamma * target q values;**23** Calculate loss function ← mean squared error (squared L2 norm) between q_values and expected_q_values;**24** Decrease epsilon ← Maximum value of either epsilon * epsilon_decay or min epsilon;**25** Increment episode counter by 1;**26** OUTPUT episode count, total reward, epsilon value;**27** **return** decision;

#### 3.3.4. Step 4—Agent Movement

Algorithm 4 outlines the procedure for agent movement using DQL. It begins by specifying the input parameters for the agent, including the input size, output size, learning rate, discount factor (gamma), exploration rate (epsilon), epsilon decay rate, minimum epsilon value, replay memory size, and batch size. The number of episodes is set to a predefined value. For each episode, the algorithm initializes the state to its initial value and sets the done flag to indicate that the episode is ongoing. The total reward is initialized to track the cumulative reward obtained in the episode. The algorithm enters a loop that iterates until the episode is done. Within this loop, the agent selects an action based on an exploration-exploitation trade-off: if the exploration rate (epsilon) is greater than a random value, the agent selects the action with the highest Q value based on the current state; otherwise, it chooses a random action. The algorithm then interacts with the environment, obtaining the next state, reward, and done flag. These values are stored in the replay memory. The total reward is updated by adding the reward obtained in the current step. The state is updated with the next state. The Q values for the current state are obtained from the prediction model, while the target Q values for the next state are obtained from the target model. The expected Q values are computed as the aggregate of the reward, the discount factor (gamma), and the target Q values. The loss function, calculated using the Mean Squared Error [22,23], measures the discrepancy between the predicted Q values and the expected Q values, allowing the agent to adjust its predictions. The exploration rate (epsilon) is decreased according to a decay rate or until it reaches the minimum value. The episode count, total reward, and epsilon value are returned as the output. The algorithm repeats until all episodes are completed, facilitating the learning and improvement of the agent’s decision-making process.   
**Algorithm 4:** Procedure for Agent Movement  **1** **Input**: input size, output size, learning rate, gamma, epsilon, epsilon decay, min epsilon, replay memory size, batch size
    **Output**: episode count, total reward, epsilon value
  **2** SET num_episodes to 1000;  **3** **for** *episode in range(num_episodes)* **do**  **4**  state ← Reset state to 0;  **5**  done ← Set done to false;  **6**  total_reward ← Set total reward to 0;  **7**  **while** *not done* **do**  **8**   Take action ← If epsilon > random value, q values = current state. ELSE random action;  **9**   Assign values for next_state, reward, done ← Take a step in the simulator;**10**   Append replay_memory ← state, action, reward, next_state, done values;**11**   Increment total_reward with reward;**12**   Update state value with the next state;**13**   SET Q Values ← Set state values to the prediction model;**14**   SET Target Q Values ← Set target model values to next_state;**15**   SET Expected Q Values ← aggregate reward * gamma * target q values;**16**   Calculate loss function ← mean squared error (squared L2 norm) between q_values and expected_q_values;**17**   Decrease epsilon ← Maximum value of either epsilon * epsilon_decay or min epsilon;**18**   OUTPUT episode count, total reward, epsilon value;**19**  **end****20** **end**

### 3.4. Performance Metrics

We evaluate the efficacy of our proposed solution using the following metrics.

#### 3.4.1. Total Delay Calculation

Algorithm 5 aims to calculate the total delay for a given set of VM containers in a cloud environment. It begins by initializing the variable total to 0, which will accumulate the total delay. It then iterates over each VM container in the VM container list. If a VM container is None, indicating an empty slot, the algorithm skips it and continues to the next iteration. For each non-empty VM container, the algorithm retrieves the relevant host and VM container information, including the associated network latency. It also calculates the computation delay of the VM container by considering the remaining steps to be executed and the apparent Instructions Per Second (IPS). The term “Apparent” is used to indicate that the IPS value is adjusted or estimated based on the observed performance characteristics of the system. This adjustment helps to provide a more realistic measure of the effective processing capacity of the system when calculating the computation delay for a VM container. The total delay of the VM container is obtained by summing the network delay and the computation delay. This total delay is then added to the total variable. Finally, after iterating through all the VM containers, the algorithm returns the accumulated value of the total, representing the overall delay experienced by the non-empty VM containers in the given cloud environment.   
**Algorithm 5:** Total Delay Calculation  **1** **Input**: VM container list, total instructions, completed instructions, apparent IPS
    **Output**: total delay
  **2** total ← 0;  **3** **for** *VM container in VM container_list* **do**  **4**  **if** *the VM container is None* **then**  **5**   continue;  **6**  **end**  **7**  network_delay_of_container ← Retrieve Host and Container by ID and relevant latency;  **8**  computation_delay_of_container ← (totalInstructions - completedInstructions) / ApparentIPS;  **9**  total_delay_of_container ← network_delay_of_container + computation_delay_of_container;**10**  total ← total_delay_of_container + total;**11** **end****12** **return** total;

#### 3.4.2. Total Power Consumption Calculation

Algorithm 6 calculates the total power consumption for a list of hosts in a cloud environment. The  SPECpower_ssj2008 [24] was used as the benchmark to represent CPU load and corresponding power utilization. It uses the left_range and right_range variables to represent the power consumption values at different utilization levels. left_range corresponds to the lower utilization level (index) in the list of power utilization, while right_range represents the higher utilization level (index + 1). The alpha variable determines the proportion of power consumption between left_range and right_range based on the fractional part of the CPU utilization divided by 10. By performing linear interpolation with alpha, the algorithm estimates the power consumption for the given CPU utilization, ensuring a smooth transition between utilization levels. The power consumption estimation is returned as the result of the power function, calculated by multiplying alpha with right_range and (1 − alpha) with left_range and then summing the two values.    
**Algorithm 6:** Total Power Consumption Calculation  **1** **Input**: host list
    **Output**: total power consumption
  **2** total ← 0;  **3** **for** *host in host_list* **do**  **4**  cpu ← Retrieve CPU usage;  **5**  power_list_index ← floor(CPU/10);  **6**  left_range ← power_list[index];  **7**  right_range ← power_list[index + 1 if cpu % 10 ≠ 0 else index];  **8**  alpha ← (CPU/10) − index;  **9**  power ← alpha * right + (1 − alpha) * left;**10**  total ← power + total;**11** **end****12** **return** total;


#### 3.4.3. Migration Cost Calculation

Algorithm 7 is a function that calculates the migration cost in a system. The algorithm takes input parameters decisions, totalBW, and env. It initializes variables total and migrationTime to 0, and routerBwToEach_Host is computed. The algorithm iterates over each container_id and corresponding new_host_id from decisions. For each VM container, it retrieves the VM container object and checks if it exists. If not, it returns 0 as the migration cost. If migration is required, it computes the migration time based on the VM container size and allocated bandwidth. Additionally, it adds the latency difference if the new host ID is valid. The migration time is added to the total, representing the cumulative migration cost. In the end, the algorithm returns the total migration cost.   
**Algorithm 7:** Migration Cost Calculation  **1** **Input**: decisions, totalBW, env
    **Output**: total migration cost
  **2** total ← 0;  **3** migrationTime ← 0;  **4** routerBwToEach_Host ← totalBW/len(decisions);  **5** **for** *VM container_id, new_host_id in decisions* **do**  **6**  numberAllocToHost ← len(MigrationToHost(new_host_id, decisions));  **7**  allocBw ← min(HostByID(new_host_id).bwCap.downlink/numberAllocToHost, routerBwToEach_Host);  **8**  VM container ← env.getContainerByID(VM container_id);  **9**  **if** *VM container is None* **then****10**   **return** 0;**11**  **end****12**  **if** *hostID ≠ new_host_id* **then****13**   migrationTime += VM containersize()/allocBw;**14**   **if** *new_host_id ≠ −1* **then****15**    migrationTime += abs(env.hostlist[hostid].latency − hostlist[new_host_id].latency);**16**   **end****17**  **end****18**  total += migrationTime;**19** **end****20** **return** total;

   Finally, the reward function is calculated as illustrated in Equation (6):

(6) $$\begin{array}{c} \mathrm{reward}(\mathrm{env},\mathrm{decision})=-(\mathrm{total}\_\mathrm{delay}(\mathrm{env})+\\ \mathrm{total}\_\mathrm{power}\_\mathrm{consumption}(\mathrm{env})+\mathrm{total}\_\mathrm{migration}\_\mathrm{cost}(\mathrm{env},\mathrm{decision})) \end{array}$$

Here, the reward function takes the environment state and decision as input. It calculates the total delay, total power consumption, and total migration cost in the given environment using corresponding functions. The resulting values are then summed together and negated to represent the negative reward. The initial weights for delay and power consumption are set to 1 to maintain a balanced consideration between these metrics. A value of 1 means that both metrics are considered equally important initially. Beyond that point, the weight values are determined by the action of the agent and the state space, which is a result of the number of containers and hosts in the system. The state space accounts for the information needed to represent the entire state of the system. The reward scaling function determines the subsequent actions, and it is configured to have a reward range from −1 to 1. Here, −1 represents a negative reward (penalty), and 1 represents a positive reward (reinforcement). This allows the agent to maximize rewards and improve its performance over time.

The overall migration cost is calculated for a list of container migration decisions made by the agent during a specific time slice. It sums up the individual migration costs for each container, considering the container’s size, migration time, and CPU utilization. This cost is essential for the agent to optimize its actions, considering the trade-offs between the benefits of moving containers to more suitable hosts and the cost associated with the migration process.

### 3.5. Experimental Setup

We conducted experiments using a simulation framework that emulates the FCC environment. The simulation framework uses the real-world workload and energy consumption data as benchmarks. We compare the performance of our proposed solution with existing solutions in the literature in the subsequent section.

As shown in Algorithm 8, we first initialize an empty list to store the generated VM containers. It then iterates over a range determined by a Gaussian distribution of the number of VM containers, ensuring a minimum of 1 VM container is generated. For each iteration, it assigns a CreationID and selects a random index. To create a realistic workload representation, we generated new VM containers based on real-world workload. The Azure 2017 workload dataset [25,26] was used as a benchmark for the simulation framework. This dataset consists of CSV files that provide information about CPU utilization in Megahertz and workload characteristics in the Azure cloud environment, recorded at intervals of 5 min for a total of 30 days. These CSV files are read as DataFrames.

SLA values are generated based on a Gaussian distribution. IPS and other metrics are calculated using the DataFrame values. Instances of different classes are created with the calculated values. The tuples containing the VM container details are appended to workloadlist and creation_id is incremented. The generated VM containers are added to generated_VM_containers. Finally, the method returns the result of a method called deployed_VM_containers. Overall, this code generates new VM containers for the workload based on various parameters and metrics, creating a diverse workload representation.
**Algorithm 8:** Experimental Setup - Generate VM Containers  **1** Initialize *generated_VM_containers* as an empty list;  **2** Generate *num_VM_containers* based on a Gaussian distribution;  **3** Ensure *num_VM_containers* is at least 1;  **4** **for** *i in range(num_VM_containers)* **do**  **5**  Assign *CreationID* to the VM container;  **6**  Select a random index;  **7**  Read CSV files as DataFrames representing the Azure 2017 workload;  **8**  Generate SLA values based on a Gaussian distribution;  **9**  Calculate IPS and other metrics using DataFrame values;**10**  Create instances of different classes with the calculated values;**11**  Append the tuple containing VM container details to *workloadlist*;**12**  Increment *creation_id*;**13**  Add the generated VM container to *generated_VM_containers* list;**14** **end****15** Return the result of *deployed_VM_containers*;

The purpose of using the Gaussian distribution in the For loop is to introduce randomness and variability in the generated VM containers [27]. The number of iterations in the For loop, which represents the number of VM containers to be generated, is determined by sampling from a Gaussian distribution. In the code, the range() function is used with a parameter of max(1, int(gauss(mean, sigma))). The gauss() function is from the random module and generates random numbers following a Gaussian distribution (also known as a normal distribution). By using the Gaussian distribution, the number of VM containers generated will tend to cluster around the mean value but with some level of randomness determined by the sigma value. The result is that the number of VM containers will vary within a range, creating a more realistic and diverse workload [28]. It lends to the idea that it is representative of other cloud environments as well. This approach allows for generating workload files with different numbers of VM containers each time the method is called, adding variability to the workload and potentially simulating real-world scenarios where the number of VM containers fluctuates.

For the DQL algorithm, the Gymnasium library was utilized [29]. We adopted a learning rate of 0.001. The learning rate determines the step size during weight updates in the neural network. A smaller learning rate helps the network make more gradual adjustments, which is crucial for achieving stable learning in complex environments. The discount factor (gamma) was set to 0.99. This parameter controls the balance between immediate and future rewards. By giving future rewards a non-zero weight, the agent is encouraged to consider the long-term consequences of its actions, promoting more strategic decision-making.

To handle the exploration-exploitation trade-off, we implemented an epsilon-greedy policy [30]. Initially, the exploration rate (epsilon) was set to 1.0, meaning the agent mostly explored the environment at the beginning of training. We chose a high initial epsilon to encourage sufficient exploration and avoid prematurely converging to suboptimal policies. Over time, the epsilon value was set to decay with a factor of 0.995, gradually reducing the agent’s exploration rate. This decay allows the agent to shift its focus towards exploiting the knowledge it acquired during training, ultimately improving the quality of its decisions.

For the minimum exploration rate (minimum epsilon), we set it to 0.01. This ensures that the agent maintains some level of exploration even after extensive training, preventing it from becoming overly deterministic and potentially missing better solutions. To facilitate efficient learning and sample diversity, we used a replay memory size of 10,000. The replay memory stores past experiences, enabling the agent to learn from a random selection of experiences rather than relying solely on the most recent ones. A large replay memory size ensures that the agent can better generalize its experiences and avoid potential biases. The batch size for updating the neural network was set to 32. A larger batch size helps the agent learn from multiple experiences simultaneously, leading to more stable and efficient learning compared to using just one experience at a time.

Overall, these parameter settings were carefully chosen to strike a balance between exploration and exploitation, optimize learning performance, and ensure convergence of the DQL algorithm.

The experiments were run on a system with an Intel i7 processor, 16 GB RAM along with Python(3.7), PyTorch(1.7.1) [31], Sci-kit learn(1.0.2) [32], Gymnaisum(0.26. 3) [29], Matplotlib(3.3.2) [33], Seaborn(0.12.2) [34], Numpy(1.19.2) [35] and Pandas (1.1.2) [36].

## 4. Results

We compare our work with popular scheduling algorithms such as Heteroscedastic Gaussian Processes (HGP) [37,38,39], Inter-Quartile Range Minimum Migration Time (IQR-MMT) [40], Median Attribute Deviation Minimum Migration Time (MAD-MMT) [41], Robust Logistic Regression Minimum Migration Time (RLR-MMT) [42] and Genetic Algorithm (GA) [43].

HGP is an auto-tuning algorithm that leverages gaussian processes to find optimal configurations for stream processing systems within a limited experimental budget. It captures posterior distributions of the configuration spaces using Bayesian Optimization. IQR-MMT is an energy-efficient approach for resource management in cloud computing. It uses the Cuckoo Optimization Algorithm (COA) to detect over-utilized hosts and employs the MMT policy to migrate VMs to achieve better resource utilization and lower energy consumption. MAD-MMT focuses on efficiently managing cloud resources through effective VM selection policies and hotspot detection mechanisms. The introduced policies, Median Migration Time, and Maximum Utilization aim to minimize energy consumption, service level agreement violations, and the number of migrations. RLR-MMT aims to optimize resource utilization and energy efficiency in cloud data centers. It proposes a Logistic Regression-based host overloading prediction technique for VM consolidation by migrating or consolidating VMs to prevent host overloading. GA is an approach for energy reduction in cloud data centers. It tackles the NP-Hard problem of container consolidation using heuristic and metaheuristic algorithms. The proposed Energy Efficient Genetic Algorithm (EEGA) attempts to optimize energy consumption and resource utilization.

**Figure 5:**
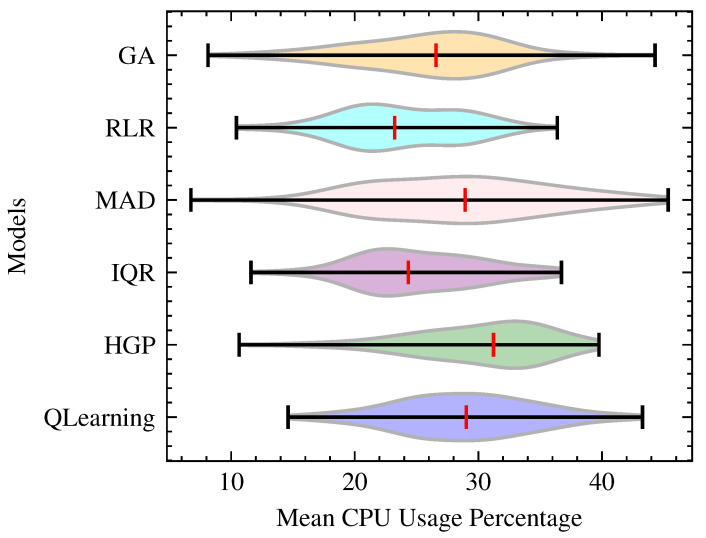
CPU Performance per Model.

**Figure 6:**
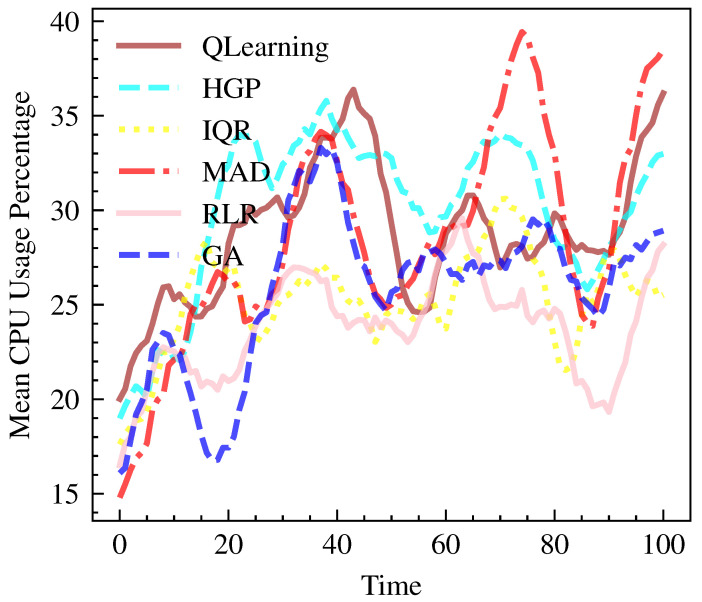
CPU Performance over time per Model.

As shown in Figure 5 and Figure 6, QLearning demonstrates efficient CPU utilization with a median value of 29.02, only second to HGP with 31.22. The remaining models, including IQR, MAD, RLR, and GA, have lower mean CPU usage percentages, ranging from 23.22 to 28.93. The Median value is taken into account, as real cloud workloads tend to have asymmetric distributions with frequent spikes [44]. If the mean is used, it would be significantly impacted by the outliers. Thus, median is robust as it less sensitive to extreme values in the context of CPU utilization [45]. With the violin plot, the central tendency of CPU utilization across different model workloads is illustrated while considering the spread and shape of the distributions.

**Figure 7:**
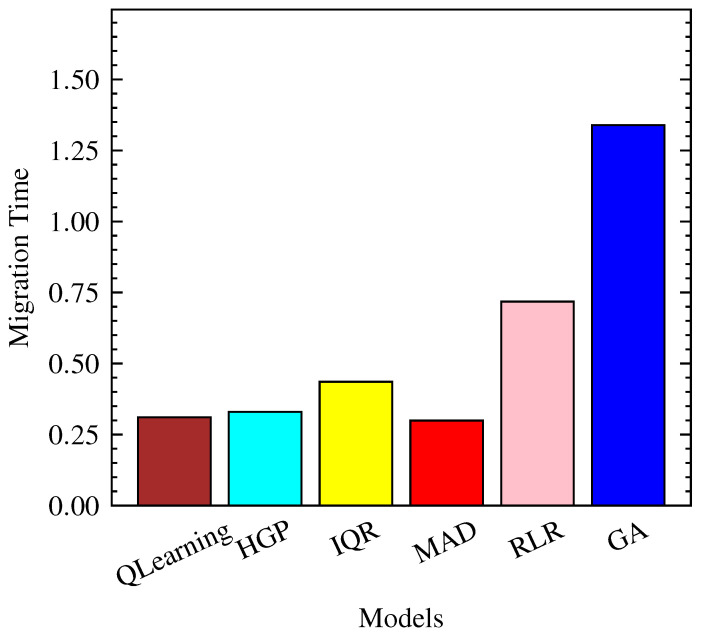
Mean Migration Time in seconds per model.

**Figure 8:**
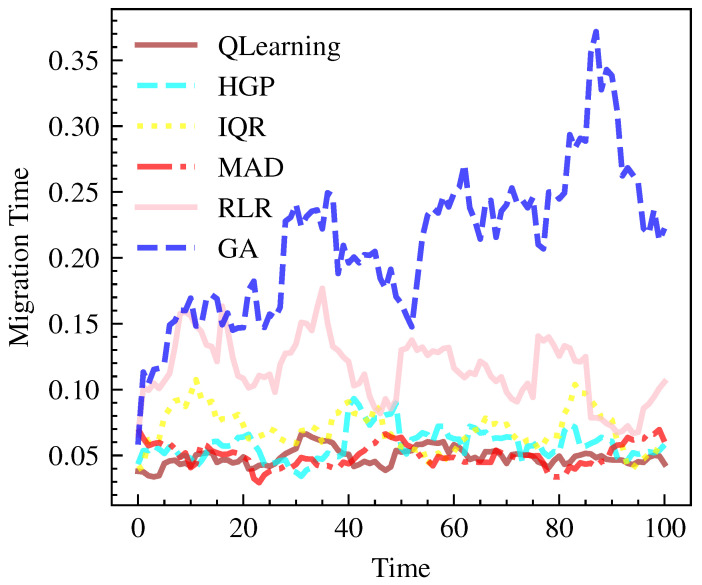
Migration Time over time per model.

QLearning demonstrates superior efficiency in terms of migration time, averaging 0.31 units with a small standard deviation, as shown in Figure 7 and Figure 8. This indicates that QLearning requires, on average, less time for migration compared to the other models. On the other hand, GA exhibits the longest migration time, with an average of 1.34 units.

**Figure 9:**
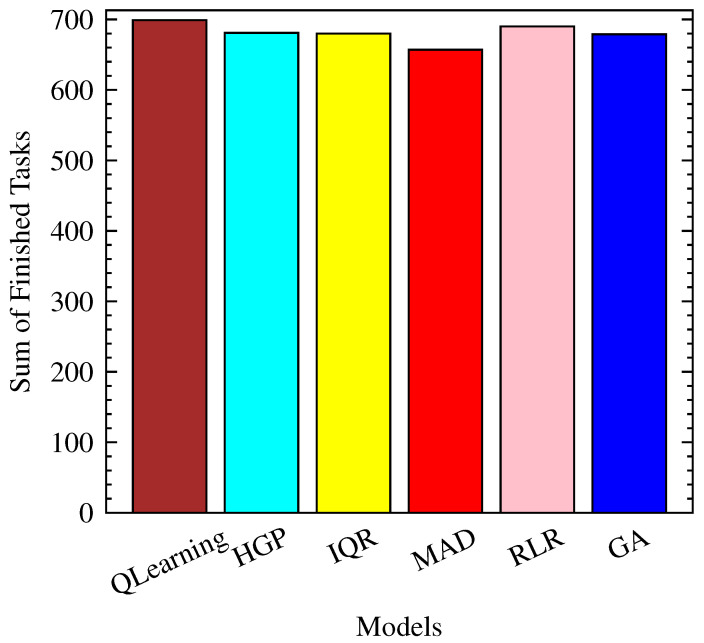
Total finished tasks per model.

**Figure 10:**
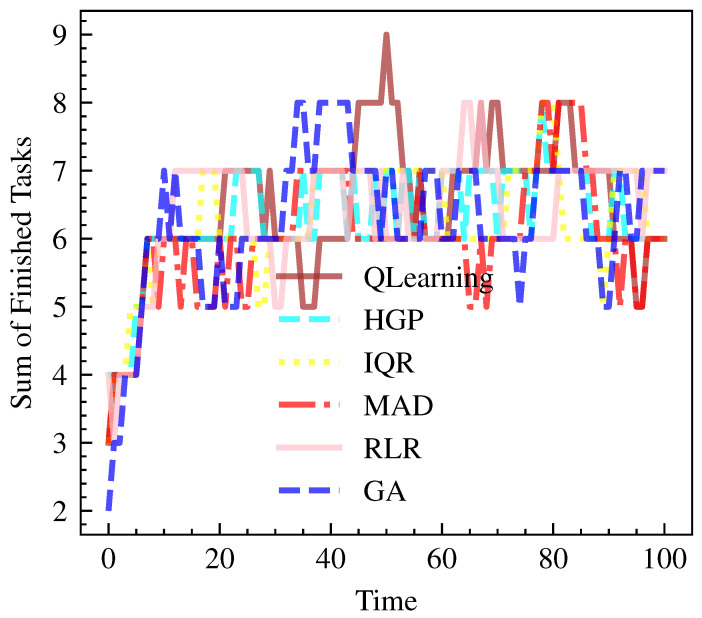
Finished tasks over time per model.

As shown in Figure 9 and Figure 10, QLearning stands out once again in terms of the number of finished tasks, with an average of 699 tasks completed. This performance is closely followed by RLR, with an average of 690 tasks. The remaining models, including HGP, IQR, MAD, and GA, achieve slightly lower numbers of finished tasks, ranging from 657 to 681.

**Figure 11:**
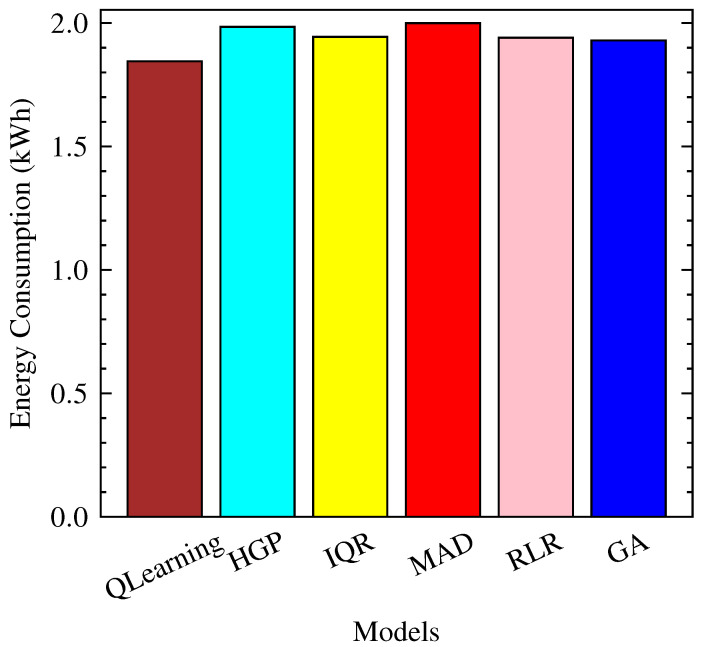
Energy consumption per Model.

Among the evaluated models in Figure 11, QLearning exhibits the lowest energy consumption, with an average of 1.85 kWh and a negligible standard deviation. This indicates that QLearning consumes the least amount of energy compared to the other models. It is worth noting that the other models, such as HGP, IQR, MAD, RLR, and GA, also have relatively similar energy consumption levels to QLearning, ranging from 1.93 kWh to 2.00 kWh.

**Figure 12:**
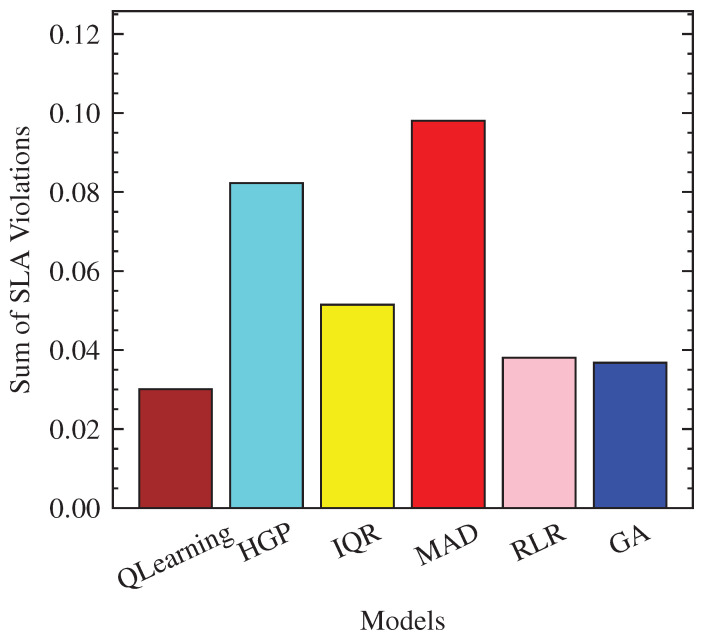
Proportionate SLA violations per model.

With Figure 12, QLearning shows notable performance by having the lowest number of SLA violations, averaging only 0.03 violations. The other models, such as HGP, IQR, MAD, RLR, and GA, have slightly higher numbers of SLA violations, ranging from 0.04 to 0.10.

In summary, the QLearning model consistently performs better than the other evaluated models across various metrics. It consumes the least amount of energy, achieves a high number of finished tasks, requires minimal migration time, experiences fewer SLA violations, and efficiently utilizes CPU resources. These findings highlight the superiority of QLearning in FCC environment scenarios and make it an attractive choice for optimizing performance.

## 5. Discussion

The results of the simulations are illustrated in Table 1

Energy Consumption: The QLearning model consumes approximately 5.5% less energy compared to the average energy consumption of the other models. This highlights QLearning’s efficiency in utilizing resources, leading to reduced energy costs and environmental impact.

Sum of Finished Tasks: QLearning achieves approximately 3.2% more finished tasks than the average number of finished tasks of the other models. This indicates that QLearning demonstrates a slightly higher capability to successfully complete tasks, showcasing its effectiveness in task management.

Migration Time: QLearning completes migrations approximately 50.5% faster than the average migration time of the other models. This substantial improvement in migration time emphasizes QLearning’s ability to swiftly adapt and transfer workloads, resulting in minimized system downtime and improved overall performance.

Sum of SLA Violations: QLearning exhibits exceptional performance by having approximately 51.6% fewer SLA violations compared to the average number of violations observed in the other models. This highlights QLearning’s reliability and adherence to service level agreements, ensuring better service quality and customer satisfaction.

Median CPU Usage Percentage: QLearning utilizes CPU resources approximately 8.1% more efficiently than the average CPU usage percentage of the other models. This suggests that QLearning optimizes the allocation and distribution of computational resources, leading to enhanced performance and resource utilization.

While QLearning may not surpass the other models in terms of the number of CPU usage efficiency, it excels in significant aspects such as energy consumption, migration time, SLA violations, and finished tasks. These strengths highlight QLearning’s potential to improve resource management, system stability, and overall efficiency in FCC environments. The significance of these findings lies in the fact that it promotes a solution that optimizes the VM placement and energy efficiency while preserving SLA agreement in FCC environments.

FCC environments are highly complex due to the involvement of multiple DCs and the need for efficient workload management across them. Traditional methods often struggle to handle the complexity and dynamic nature of these environments. FEDQWP handles complex and multi-dimensional scenarios by using explorative techniques as well as neural networks. This makes it well-suited for addressing the challenges posed by FCC environments. One of the key strengths of FEDQWP is its ability to learn from past experiences through a trial-and-error approach. By continuously interacting with the environment and optimizing its actions based on rewards and penalties, the DQL model gradually improves its decision-making capabilities, leading to better performance over time. Other methods rely on extensive training on historical data, while the explorative nature of our solution can make fast decisions without relying as much on historical data. Another advantage is the consideration of multiple important metrics, including energy consumption, finished tasks, migration time, SLA violations, and CPU usage. These factors collectively make the DQL model a comprehensive and effective solution for workload prediction in FCC environments.

While the proposed FEDQWP method shows promising performance, there are a few potential limitations to consider. We evaluate FEDQWP’s performance using real-world workloads, but the generalizability of the model to a wide range of FCC environments should be considered. The performance of the DQL model may vary when applied to different data center architectures, workload characteristics, and resource allocation policies. Changes in workload patterns or infrastructure dynamics over time may require continuous retraining of the model to maintain its performance. This might involve having to tune various hyperparameters, such as the learning rate, discount factor, and exploration rate, to achieve optimal results.

## 6. Conclusions

In this study, we proposed FEDQWP, a novel approach to address the challenges of energy and SLA-aware workload prediction and VM scheduling in geographically distributed FCC environments using DQL. Our proposed approach demonstrated significant improvements over the existing methods in terms of energy efficiency and SLA compliance. The use of DQL in this study opens up exciting possibilities for the optimization of FCC resources in the future. We can thus continue to improve the efficiency, sustainability, and scalability of FCC by leveraging the power of ML and supporting the growth of the futuristic economy.

## 7. Future Work

Overall, our proposed approach provides a comprehensive and effective solution to the challenges faced by CSPs in geographically distributed FCC environments. Future research could explore the application of our approach to different types of workloads, such as real-time applications or big data processing, and investigate the potential of combining our approach with other optimization techniques to further improve its performance.

## Figures and Tables

**Table 1:** Performance Metrics of Cloud Computing Models.

Models	Energy Consumption (kWh)	Sum of Finished Tasks	Migration Time	Sum of SLA Violations	Median CPU Usage Percentage
QLearning	1.85 ± 0.00	699.00 ± 0.00	0.31 ± 0.03	0.03 ± 0.00	29.02 ± 0.94
HGP	1.98 ± 0.00	681.00 ± 0.00	0.33 ± 0.03	0.08 ± 0.00	31.22 ± 0.92
IQR	1.94 ± 0.00	680.00 ± 0.00	0.44 ± 0.05	0.05 ± 0.00	24.33 ± 0.84
MAD	2.00 ± 0.00	657.00 ± 0.00	0.30 ± 0.03	0.10 ± 0.00	28.93 ± 1.21
RLR	1.94 ± 0.00	690.00 ± 0.00	0.72 ± 0.08	0.04 ± 0.00	23.22 ± 0.84
GA	1.93 ± 0.00	679.00 ± 0.00	1.34 ± 0.14	0.04 ± 0.00	26.58 ± 1.02

## Data Availability

Publicly available datasets were analyzed in this study. This data can be found here: https://github.com/Azure/AzurePublicDataset/blob/master/AzurePublicDatasetV1.md (AzurePublicDatasetV1, accessed on 6 February 2023).

## Abbreviations

The following abbreviations are used in this manuscript:

FEDQWP	Federated Cloud Workload Prediction with Deep Q-Learning
FCC	Federated Cloud Computing
CSP	Cloud Service Provider
SLA	Service Level Agreement
DQL	Deep Q Learning
VM	Virtual Machine
DC	Data Center
SaaS	Software-as-a-Service
PaaS	Platform-as-a-Service
IaaS	Infrastructure-as-a-Service
DBN	Deep Belief Network
CPU	Central Processing Unit
RC	Resource Collector
RBM	Restricted Boltzmann Machines
MMT	Minimum Migration Time
PBC	Prototype-Based Clustering
DBC	Density-based clustering
SGW	Savitzky–Golay and Wavelet-supported
SCN	Stochastic Configuration Networks
DRL	Deep Reinforcement Learning
LSTM	Long Short Term Memory
RNN	Recurrent Neural Network
IPS	Instructions per Second
DL	Deep Learning
RM	Resource Manager
NN	Neural Network
ML	Machine Learning
HGP	Heteroscedastic Gaussian Processes
COA	Cuckoo Optimization Algorithm
IQR	Inter-Quartile Range
MAD	Median Attribute Deviation
RLR	Robust Logistic Regression
GA	Genetic Algorithm
EEGA	Energy Efficient Genetic Algorithm

## References

[1] Petrovska I., Kuchuk H., Mozhaiev M. (2022). Features of the distribution of computing resources in cloud systems. Proceedings of the 2022 IEEE 3rd KhPI Week on Advanced Technology (KhPIWeek).

[2] Angel N.A., Ravindran D., Vincent P.D.R., Srinivasan K., Hu Y.C. (2021). Recent advances in evolving computing paradigms: Cloud, edge, and fog technologies. Sensors.

[3] Alkhamees S. (2022). SLA Negotiation and Renegotiation in Cloud SLA Management: Issue and Challenges. Proceedings of the IoT as a Service: 7th EAI International Conference, IoTaaS 2021.

[4] ITU (2014). impl. 09.03. 2014.

[5] Toosi A.N., Calheiros R.N., Buyya R. (2014). Interconnected cloud computing environments: Challenges, taxonomy, and survey. ACM Comput. Surv..

[6] De Bruin B., Floridi L. (2017). The ethics of cloud computing. Sci. Eng. Ethics.

[7] Schleier-Smith J., Sreekanti V., Khandelwal A., Carreira J., Yadwadkar N.J., Popa R.A., Gonzalez J.E., Stoica I., Patterson D.A. (2021). What serverless computing is and should become: The next phase of cloud computing. Commun. ACM.

[8] Akintoye S.B., Bagula A. (2019). Improving quality-of-service in cloud/fog computing through efficient resource allocation. Sensors.

[9] Han G., Que W., Jia G., Shu L. (2016). An efficient virtual machine consolidation scheme for multimedia cloud computing. Sensors.

[10] Qiu F., Zhang B., Guo J. (2016). A deep learning approach for VM workload prediction in the cloud. Proceedings of the 2016 17th IEEE/ACIS International Conference on Software Engineering, Artificial Intelligence, Networking and Parallel/Distributed Computing (SNPD).

[11] Ruan L., Bai Y., Li S., He S., Xiao L. (2023). Workload time series prediction in storage systems: A deep learning based approach. Clust. Comput..

[12] Nagpure M.B., Dahiwale P., Marbate P. (2015). An efficient dynamic resource allocation strategy for VM environment in cloud. Proceedings of the 2015 International Conference on Pervasive Computing (ICPC).

[13] Jassas M.S., Mahmoud Q.H. (2022). Analysis of Job Failure and Prediction Model for Cloud Computing Using Machine Learning. Sensors.

[14] Beloglazov A., Buyya R. (2012). Optimal online deterministic algorithms and adaptive heuristics for energy and performance efficient dynamic consolidation of virtual machines in cloud data centers. Concurr. Comput. Pract. Exp..

[15] Kumar J., Goomer R., Singh A.K. (2018). Long short term memory recurrent neural network (LSTM-RNN) based workload forecasting model for cloud datacenters. Procedia Comput. Sci..

[16] Zhu Y., Zhang W., Chen Y., Gao H. (2019). A novel approach to workload prediction using attention-based LSTM encoder-decoder network in cloud environment. EURASIP J. Wirel. Commun. Netw..

[17] Gao J., Wang H., Shen H. (2020). Machine learning based workload prediction in cloud computing. Proceedings of the 2020 29th International Conference on Computer Communications and Networks (ICCCN).

[18] Bi J., Yuan H., Zhang L., Zhang J. (2019). SGW-SCN: An integrated machine learning approach for workload forecasting in geo-distributed cloud data centers. Inf. Sci..

[19] Chen Z., Hu J., Min G., Zomaya A.Y., El-Ghazawi T. (2019). Towards accurate prediction for high-dimensional and highly-variable cloud workloads with deep learning. IEEE Trans. Parallel Distrib. Syst..

[20] François-Lavet V., Henderson P., Islam R., Bellemare M.G., Pineau J. (2018). An introduction to deep reinforcement learning. Found. Trends Mach. Learn..

[21] Dubey S.R., Singh S.K., Chaudhuri B.B. (2022). Activation functions in deep learning: A comprehensive survey and benchmark. Neurocomputing.

[22] Sammut C., Webb G.I. (2010). Mean squared error. Encyclopedia of Machine Learning.

[23] Foundation P. Pytorch NN MSELoss. https://pytorch.org/docs/stable/generated/torch.nn.MSELoss.html#torch.nn.MSELoss.

[24] Schmitt N., Lange K.D., Sharma S., Rawtani N., Ponder C., Kounev S. The SPECpowerNext Benchmark Suite, its Implementation and New Workloads from a Developer’s Perspective. Proceedings of the ACM/SPEC International Conference on Performance Engineering.

[25] Cortez E., Bonde A., Muzio A., Russinovich M., Fontoura M., Bianchini R. Resource central: Understanding and predicting workloads for improved resource management in large cloud platforms. Proceedings of the 26th Symposium on Operating Systems Principles.

[26] Microsoft Microsoft Azure Dataset 2017. https://github.com/Azure/AzurePublicDataset/blob/master/AzurePublicDatasetV1.md.

[27] Patel E., Kushwaha D.S. (2020). Clustering cloud workloads: K-means vs gaussian mixture model. Procedia Comput. Sci..

[28] Eeckhout L., Sundareswara R., Yi J., Lilja D.J., Schrater P. (2005). Accurate statistical approaches for generating representative workload compositions. Proceedings of the IEEE International Workload Characterization Symposium.

[29] Towers M., Terry J.K., Kwiatkowski A., Balis J.U., Cola G.D., Deleu T., Goulão M., Kallinteris A., KG A., Krimmel M. (2023). Gymnasium (Version v0.26.3). Zenodo.

[30] Liu F., Viano L., Cevher V. (2022). Understanding deep neural function approximation in reinforcement learning via *ϵ*-greedy exploration. Adv. Neural Inf. Process. Syst..

[31] Paszke A., Gross S., Massa F., Lerer A., Bradbury J., Chanan G., Killeen T., Lin Z., Gimelshein N., Antiga L., Wallach H., Larochelle H., Beygelzimer A., d’Alché Buc F., Fox E., Garnett R. (2019). PyTorch: An Imperative Style, High-Performance Deep Learning Library. Proceedings of the Advances in Neural Information Processing Systems 32.

[32] Pedregosa F., Varoquaux G., Gramfort A., Michel V., Thirion B., Grisel O., Blondel M., Prettenhofer P., Weiss R., Dubourg V. (2011). Scikit-learn: Machine Learning in Python. J. Mach. Learn. Res..

[33] Hunter J.D. (2007). Matplotlib: A 2D graphics environment. Comput. Sci. Eng..

[34] Waskom M.L. (2021). seaborn: Statistical data visualization. J. Open Source Softw..

[35] Harris C.R., Millman K.J., van der Walt S.J., Gommers R., Virtanen P., Cournapeau D., Wieser E., Taylor J., Berg S., Smith N.J. (2020). Array programming with NumPy. Nature.

[36] McKinney W. (2011). pandas: A foundational Python library for data analysis and statistics. Python High Perform. Sci. Comput..

[37] Jamshidi P., Casale G. (2016). An uncertainty-aware approach to optimal configuration of stream processing systems. Proceedings of the 2016 IEEE 24th International Symposium on Modeling, Analysis and Simulation of Computer and Telecommunication Systems (MASCOTS).

[38] Bui D.M., Yoon Y., Huh E.N., Jun S., Lee S. (2017). Energy efficiency for cloud computing system based on predictive optimization. J. Parallel Distrib. Comput..

[39] Zinnen A., Engel T. (2011). Deadline constrained scheduling in hybrid clouds with Gaussian processes. Proceedings of the 2011 International Conference on High Performance Computing & Simulation.

[40] Yakhchi M., Ghafari S.M., Yakhchi S., Fazeli M., Patooghi A. (2015). Proposing a load balancing method based on Cuckoo Optimization Algorithm for energy management in cloud computing infrastructures. Proceedings of the 2015 6th International Conference on Modeling, Simulation, and Applied Optimization (ICMSAO).

[41] Sohrabi S., Moser I. (2015). The effects of hotspot detection and virtual machine migration policies on energy consumption and service levels in the cloud. Procedia Comput. Sci..

[42] Issa M.B., Daraghmeh M., Jararweh Y., Al-Ayyoub M., Alsmirat M., Benkhelifa E. (2017). Using logistic regression to improve virtual machines management in cloud computing systems. Proceedings of the 2017 IEEE 14th International Conference on Mobile Ad Hoc and Sensor Systems (MASS).

[43] Patel D., Patra M.K., Sahoo B. (2020). Energy efficient genetic algorithm for container consolidation in cloud system. Proceedings of the 2020 7th International Conference on Signal Processing and Integrated Networks (SPIN).

[44] Bao L., Yang J., Zhang Z., Liu W., Chen J., Wu C. (2023). On accurate prediction of cloud workloads with adaptive pattern mining. J. Supercomput..

[45] Khorana A., Pareek A., Ollivier M., Madjarova S.J., Kunze K.N., Nwachukwu B.U., Karlsson J., Marigi E.M., Williams R.J. (2023). Choosing the appropriate measure of central tendency: Mean, median, or mode?. Knee Surg. Sport. Traumatol. Arthrosc..

